# *ZmPP2C26* Alternative Splicing Variants Negatively Regulate Drought Tolerance in Maize

**DOI:** 10.3389/fpls.2022.851531

**Published:** 2022-04-08

**Authors:** Fengzhong Lu, Wanchen Li, Yalin Peng, Yang Cao, Jingtao Qu, Fuai Sun, Qingqing Yang, Yanli Lu, Xuehai Zhang, Lanjie Zheng, Fengling Fu, Haoqiang Yu

**Affiliations:** ^1^Key Laboratory of Biology and Genetic Improvement of Maize in Southwest Region, Ministry of Agriculture, Maize Research Institute, Sichuan Agricultural University, Chengdu, China; ^2^National Key Laboratory of Wheat and Maize Crop Science, Henan Agricultural University, Zhengzhou, China

**Keywords:** maize, drought stress, protein phosphatase 2C, MAPK, alternative splicing, photosynthesis

## Abstract

Serine/threonine protein phosphatase 2C (PP2C) dephosphorylates proteins and plays crucial roles in plant growth, development, and stress response. In this study, we characterized a clade B member of maize PP2C family, i.e., ZmPP2C26, that negatively regulated drought tolerance by dephosphorylating ZmMAPK3 and ZmMAPK7 in maize. The *ZmPP2C26* gene generated *ZmPP2C26L* and *ZmPP2C26S* isoforms through untypical alternative splicing. ZmPP2C26S lost 71 amino acids including an MAPK interaction motif and showed higher phosphatase activity than ZmPP2C26L. ZmPP2C26L directly interacted with, dephosphorylated ZmMAPK3 and ZmMAPK7, and localized in chloroplast and nucleus, but ZmPP2C26S only dephosphorylated ZmMAPK3 and localized in cytosol and nucleus. The expression of *ZmPP2C26L* and *ZmPP2C26* was significantly inhibited by drought stress. Meanwhile, the maize *zmpp2c26* mutant exhibited enhancement of drought tolerance with higher root length, root weight, chlorophyll content, and photosynthetic rate compared with wild type. However, overexpression of *ZmPP2C26L* and *ZmPP2C26S* significantly decreased drought tolerance in *Arabidopsis* and rice with lower root length, chlorophyll content, and photosynthetic rate. Phosphoproteomic analysis revealed that the ZmPP2C26 protein also altered phosphorylation level of proteins involved in photosynthesis. This study provides insights into understanding the mechanism of PP2C in response to abiotic stress.

## Introduction

In plants, numerous proteins will be activated or inactivated *via* dephosphorylation catalyzed by protein phosphatases (PPs) (Cohen, 1989). Based on their substrate specificity, PPs are mainly classified into three families, namely, serine (Ser)/threonine (Thr)-specific phosphoprotein phosphatase (PPP), metal-dependent protein phosphatase (PPM), and protein tyrosine phosphatase (PTP) (Barford et al., 1998). The PPP and PPM families encode Ser/Thr PP, while PTP family includes tyrosine-specific and dual-specificity phosphatase (Barford et al., 1998). The PP2C of PPM family is a kind of the Mg^2+^- or Mn^2+^-dependent PPs and specifically dephosphorylates the phosphorylated Ser/Thr residues of target proteins (Schweighofer et al., 2004; Shi, 2009). The PP2C family is substantially expanded in plants with 80, 90, and 130 members in *Arabidopsis*, rice, and maize, respectively, and divided into eleven clades of A–K (Xue et al., 2008; Singh et al., 2010; Wang et al., 2018). The clade A members of PP2C act as co-receptors of abscisic acid (ABA), interact with the ABA-receptor protein PYR/PYL/PCAR and SNF1-related protein kinase 2s (SnRK2s) to negatively regulate ABA signaling, and play crucial roles in plant growth, development, and stimuli response (Ma et al., 2009; Park et al., 2009; Komatsu et al., 2013). For instance, AHG1 encoding a PP2C interacts with DELAY OF GERMINATION1 (DOG1) and is impaired by DOG1 to negatively regulate ABA response in seed dormancy and germination (Nishimura et al., 2018). The maize *ZmPP2C-A2*, *ZmPP2C-A6*, and *ZmPP2C-A10* negatively regulate drought tolerance by meditating ABA signaling (Xiang et al., 2017; He et al., 2019). Tomato *SlPP2C3* functions as a negative regulator of ABA signaling to negatively regulate drought tolerance, fruit ripening, and glossiness (Liang et al., 2021). In contrast, only few available studies report the function of other clade PP2Cs. A clade G member *AtPP2C49* negatively regulates salt tolerance through inhibition of Na^+^ transporter AtHKT1;1 activity (Chu et al., 2021). In *Arabidopsis*, three of six clade B members of PP2C including *AP2C1*, *AP2C3*, and *PP2C5* are well-elucidated for their function in stomata development, immunity, defense, and K^+^ deficiency response (Schweighofer et al., 2007; Brock et al., 2010; Umbrasaite et al., 2010; Shubchynskyy et al., 2017; Singh et al., 2018). The tobacco B clade *NtPP2C2b* is found to regulate nicotine biosynthesis (Liu et al., 2021). However, the function of clade B members of PP2C in crops remains unknown.

Alternative splicing (AS) of precursor messenger RNAs (pre-mRNAs) produces more mRNA isoforms from the same pre-mRNA and plays a key role in gene expression and protein diversity (Kelemen et al., 2013). Previous studies show that abundant genes undergo AS to regulate plant growth and stress response, including salt, heat, cold, and drought stress, and photomorphogenesis, flowering, and yield (Farquharson, 2016; Jiang et al., 2017; Qin et al., 2017; Calixto et al., 2018; Gu et al., 2018; Liu et al., 2018; Yu et al., 2018; Li et al., 2019). The AS events likewise can be triggered in different environmental conditions and developmental stages (Liu et al., 2018; Martin et al., 2021). As well known, there are two typical types of intron retention, namely, U2 and U12 type with 5′-GT● ● ● ● ● ●AG-3′ and 5′-AT● ● ● ● ● ●AC-3′ splice site, respectively (Sharp and Burge, 1997; Reddy et al., 2013). Intriguingly, two untypical types of AS are recently found in *Arabidopsis*, containing alternative first exon (AFE) and alternative last exon (ALE) (Zhu et al., 2017). The *FT2* gene undergoes AFE-type AS producing *FT2*β and *FT2*α transcripts to control flowering through regulating their amount during reproductive stage (Qin et al., 2017).

Maize is one of the most important crops and is used in food supply, livestock feed, and industries. Its productivity is seriously restricted by drought stress due to its vulnerability to water deficits (Lobell et al., 2014; Sah et al., 2020). Hence, exploring stress-related genes will contribute for facilitating molecular design breeding to improve maize drought tolerance. In our previous studies, a new maize PP2C was found to be clade B member, named as *ZmPP2C26* and inhibited by drought stress (Wang et al., 2018; Lu et al., 2020), indicating its potential role in drought response. In this study, we characterized that *ZmPP2C26* underwent AFE-type AS and generated two isoforms named as *ZmPP2C26L* and *ZmPP2C26S*. Subsequently, their activity, localization, and interacting proteins were analyzed. Their functions in drought tolerance were identified through phenotyping transgenic *Arabidopsis* and rice, as well as *via* maize mutant. Our data clearly demonstrates that *ZmPP2C26* negatively regulates drought tolerance *via* dephosphorylating ZmMAPK3/ZmMAPK7 and impairing photosynthesis in maize.

## Materials and Methods

### Stress Treatment and Expression Analysis

The drought-tolerant maize inbred lines 81565/87-1 and drought-sensitive lines 200B/DAN340 were used for gene expression analysis. The four-leaf stage seedings were treated with 16% (w/v) polyethylene glycol 6000 (PEG-6000). At 0 (blank control), 3, 6, 12, and 24 h of treatment, the seedling of every line was collected and used for total RNA extraction by using RNAiso plus kit (TaKaRa, Japan).

Total RNA samples were reverse-transcribed into cDNA using PrimeScript™ reagent kit (TaKaRa) and used for real-time quantitative PCR (RT-qPCR). The RT-qPCR was performed using ChamQ Universal SYBR qPCR Master Mix (Vazyme, Nanjing) in the CFX96™ Real-Time System (Bio-Rad, Hercules, CA, United States). The *ZmEF1a* gene was used as internal control. The information of all primers used in this study is shown in Supplementary Table 1.

### Phosphatase Activity Assay

The open reading frame (ORF) of *ZmPP2C26L*/*ZmPP2C26S* without stop codon was amplified and inserted into pET32a vector to generate *His-ZmPP2C26L*/*His-ZmPP2C26S* plasmid, respectively. The reconstructed plasmids were transformed into *Escherichia coli* strain BL21. The *E. coli* strain harboring the above plasmid was induced using 0.1 mM isopropyl-1-thio-b-D-galactopyranoside (IPTG) at 16°C for 16 h to express His-ZmPP2C26L/His-ZmPP2C26S protein, which was purified using by 6 × His-Tagged Protein Purification Kit (CWBIO, China).

Protein phosphatase activity of ZmPP2C26L/ZmPP2C26S protein was detected as previously reported (Han et al., 2018; Yang et al., 2020). Briefly, 2 mg of His-tagged protein was incubated with 1 mL assay buffer (50 mM Tris–HCl, pH 7.5, 1 mM MgCl_2_, 0.5 mM EDTA, and 0.1 g/L BSA) for 30 min at 37°C. Subsequently, 2 mM p-nitrophenyl phosphate (pNPP) was added into the above mixture to produce p-nitrophenol catalyzed by ZmPP2C26L/ZmPP2C26S protein. Then, the absorbance value at wavelength of 405 nm (OD_405_) of p-nitrophenol was monitored every 2 min. The relative PP activity was calculated according to the curve of OD_405_.

### Yeast Two-Hybrid, Bimolecular Fluorescence Complementation, and Glutathione-*S*-Transferase Pull-Down

Yeast two-hybrid (Y2H) assay was conducted using the Matchmaker GAL4 Y2H System (Clontech). The ORF of *ZmPP2C26L*/*ZmPP2C26S* was amplified and inserted into prey vector pGADT7 to generate *AD*-*ZmPP2C26L*/*AD-ZmPP2C26S* plasmid, respectively. The ORF of 13 maize *PYL* genes was inserted into bait vector pGBKT7 for *BD-ZmPYLs* in our previous study (Wang et al., 2018). The ORF of 20 maize *MAPK* genes (e.g., *ZmMAPK1-19* and *ZmSIMK*) was also inserted into pGBKT7 for *BD-ZmMAPKs*, which were kindly provided by Dongtao Ren (China Agriculture University, Chengdu, China). The prey AD plasmid and the bait BD plasmid were cotransformed into *Saccharomyces cerevisiae* strain Y2H gold by using the yeast transformation kit (Coolaber, Beijing, China). The transformants were cultured on synthetic medium plates (SD medium) lacking Trp and Leu (SD/-Trp/-Leu) at 30°C for 2–3 days, then transferred onto SD/-Trp/-Leu/-His/-Ade plates containing 5-bromo-4-chloro-3-indolyl-α-D-galactopyranoside (X-α-gal) for blue color development to detect the interaction between ZmPP2C26L/ZmPP2C26S and ZmPYLs/ZmMAPKs. The interaction was further validated by bimolecular fluorescence complementation (BiFC) and glutathione-*S*-transferase (GST) pull-down assays.

For BiFC assay, the ORF of *ZmPP2C26L/ZmPP2C26S* was cloned into the pSPYNE-*35S*-*nYFP* vector generating *nYFP-ZmPP2C26L*/*nYFP-ZmPP2C26S*, respectively. The ORF of the *ZmMAPK3*/*ZmMAPK7* gene was inserted into the pSPYNE-*35S*-*cYFP* vector generating *cYFP-ZmMAPK3*/*cYFP-ZmMAPK7*, respectively. As previously described (Li et al., 2016), the *nYFP-ZmPP2C26L*/*nYFP-ZmPP2C26S* and *cYFP-ZmMAPK3*/*cYFP-ZmMAPK7* constructs were cotransformed into maize protoplast. After 16 h at 28°C, the protoplasts were examined for YFP fluorescence under the confocal laser scanning microscope (ZESS 800, Germany).

For GST pull-down assay, the ORF of *ZmMAPK3*/*ZmMAPK7* was inserted into pGEX-6p-1 vector to create *GST-ZmMAPK3*/*GST-ZmMAPK7*, respectively. The GST-tagged protein was induced by 0.1-mM IPTG and purified using Glutathione-Sepharose Resin kit (CWBIO). A total of 2 μg GST-tagged protein was uploaded to Mag-Beads GST Fusion Protein Purification (Sangon Biotech, Shanghai, China) and incubated at room temperature for 2 h. Then, 2 μg of His-tagged protein was added to protein-beads complex for combination. The protein complex was pulled down by washing five times with the elution buffer containing 50 mM Tris–HCl and 10 mM reduced glutathione (pH 8.0). The proteins were separated by 12.5% SDS-PAGE and transferred onto the PVDF (polyvinylidene fluoride) membrane by wet transfer at 100 V for 80 min. The membrane was blocked in 2.5% (w/v) non-fat milk powder solution (Coolaber, Beijing, China) for 90 min and incubated with primary antibody (anti-GST/anti-His antibody) for 90 min at room temperature and then with the HRP (horseradish peroxidase)-conjugated Goat Anti-Mouse IgG (ABclonal, Wuhan, China) for 60 min at room temperature. Finally, the signal was visualized using the ChemDoc XRS system (Bio-Rad, Hercules, CA, United States).

### Subcellular Localization and Co-localization

For subcellular localization, the ORF of *ZmPP2C26L/ZmPP2C26S* without stop codon was amplified and independently inserted into pCAMBIA2300-*35S*-*eGFP* vector to create *35S*-*ZmPP2C26L-eGFP/35S*-*ZmPP2C26S-eGFP* plasmid, respectively. The construct was transformed into *Agrobacterium tumefaciens* strain GV3101 and then used for infiltrating into the leaves of 5-week-old *Nicotiana benthamiana*. The GFP fluorescence was observed using the confocal laser scanning microscope (ZESS 800). For co-localization of ZmPP2C26L/ZmPP2C26S and ZmMAPK3/ZmMAPK7, the ORF of *ZmMAPK3*/*ZmMAPK7* was separately cloned into the pCAMBIA1300-*35S*-*mCherry* vector to generate *35S*-*ZmMAPK3*-*mCherry*/*35S*-*ZmMAPK7*-*mCherry*, respectively. The cultures of *Agrobacterium* carrying *35S*-*ZmPP2C26L*-*eGFP*/*35S*-*ZmPP2C26S*-*eGFP* and *35S*-*ZmMAPK3*-*mCherry*/*35S*-*ZmMAPK7*-*mCherry* were co-infiltrated into *N. benthamiana* leaves. The GFP and mCherry fluorescence were observed by using the confocal laser scanning microscope (ZESS 800).

### Phosphorylation Assay

The phosphorylation assay was performed with minor modification as previously described (Xia et al., 2021). The ORF of *ZmMAPK3*/*ZmMAPK7* was inserted into pCAMBIA1300-*35S*-*3* × *HA* vector to generate *35S*-*HA-ZmMAPK3/35S*-*HA-ZmMAPK7*, respectively. The constructs were transformed into maize protoplast. After transformation, the protoplasts were cultured for 16 h at 28°C. Subsequently, the total protein was extracted using total plant protein extraction kit (Coolaber, Beijing, China) and immunoprecipitated with Anti-HA Affinity Beads (Smart Lifesciences, Changzhou, China) in a rotary mixer for 4 h at 4°C. Then, 1 μg HA-tagged protein mixed with 0.25, 0.5, and 1 μg His-ZmPP2C26L/His-ZmPP2C26S protein, respectively, in the presence of 50 mM ATP and 30 μl kinase buffer (20 mM HEPES [*N*-2-hydroxyethylpiperazine-*N*-2-ethane sulfonic acid], 10 mM MgCl_2_, and 1 mM DTT, pH 7.5), and incubated for 60 min at 30°C. The reaction was stopped by adding SDS-loading buffer and separated by 12.5% phos-tag™ (Wako, Beijing, China) SDS-PAGE and normal SDS-PAGE for immunoblotting using anti-HA and anti-His antibody (ABclonal, Wuhan, China) as previously. The relative density of each band was analyzed using ImageJ software.^1^

### Phenotyping of Transgenic *Arabidopsis*, Rice, and Maize Mutant

The T-DNA insertion mutant of *ZmPP2C26* homolog *ap2c1* (SALK_065126) was obtained from the *Arabidopsis* Biological Resource Center (ABRC, Columbus, OH, United States). The *35S*-*ZmPP2C26L-eGFP/35S*-*ZmPP2C26S-eGFP* construct was transformed into *ap2c1* and Col-0 wild type (WT) for complementation and overexpression, respectively, by the floral-dip method (Clough and Bent, 1998). The seeds of homozygous lines were screened by 50 mg/L kanamycin on 1/2 MS plates without separation, planted in the soil, and cultured in greenhouse under optimal condition. The 4-week-old seedlings were kept under water deprivation for 3 weeks, then re-watered with a recovery time of 2 days. The untransformed WT and *ap2c1* line were used as control.

For transgenic rice, embryonic calli were isolated from the japonica rice variety *Nipponbare*, separately transformed by *35S*-*ZmPP2C26L* and *35S*-*ZmPP2C26S* using *Agrobacterium*-mediated transformation, screened on 1/2 MS plates containing 50 μg/ml hygromycin, regenerated, and identified by PCR. The seeds of homozygous lines and WT were germinated for 7 days. Then, 30 seedlings were transplanted into the rectangular plastic pots with mud and grown in greenhouse under a photoperiod of 14 h light 30°C/10 h dark at 25°C. Three-week-old seedings were subjected to drought treatment by withholding watering for 2 weeks, then re-watered for 3 days and photographed. The survival rate of every line was calculated. Before treatment, the photosynthetic rate of every line was measured using LI-6400XT portable photosynthesis system (LI-COR, Lincoln, NE, United States). The content of total chlorophyll was detected as previously described (Zhang et al., 2021). Meanwhile, 30 seedlings were transferred into plastic net pots, cultured in 20% PEG solution for 3 days, and measured their root length.

A maize *zmpp2c26* mutant generated by *Mu* transposon insertion within first exon was obtained from ChinaMu.^2^ It is verified by PCR and RT-PCR. The seeds of homozygous mutant were grown in soil under 28°C under a photoperiod of 14 h light/10 h dark. The three-leaf stage seedlings were subjected to drought stress by withholding watering for 3 weeks, then re-watered for 5 days and photographed. The survival rate, root length, and root dry weight of every line were measured. Before treatment, the photosynthetic rate and the content of total chlorophyll were detected as earlier. The WT isolated from heterozygous *zmpp2c26* mutant was used as control.

### Tandem Mass Tag Based Quantitative Phosphoproteomics

Rice fresh shoots of L1, S1, and Nip, as well as maize *zmpp2c26* mutant with WT were used for phosphoproteomic analysis with three biological replicates. The total protein of every line was extracted using total plant protein extraction kit (Coolaber, Beijing, China) according to the manufacturer’s instruction and quantified through BCA assay (Smith et al., 1985). For each sample, 200 μg protein solution was mixed with 5 mM DTT and 10 mM iodotyrosine in the dark at room temperature for 15 min, subsequently precipitated by adding six volumes of acetone at -20°C overnight, then centrifuged at 8,000 *g* for 10 min at 4°C to collect the precipitate, and placed for 3 min at room temperature to volatilize the acetone. After removing supernatant, 100 μl of TEAB (200 mM) was added into tube to re-dissolve the protein. Subsequently, 1/50 of the sample weight of 1 mg/ml trypsin-TPCK was added into solution and digested at 37°C overnight to generate peptides. The peptides were labeled using TMT labeling kit (Thermo Scientific, Waltham, MA, United States) and used for enriching phosphopeptides using the IMAC Phosphopeptide enrichment kit (Thermo Fisher Scientific, United States) according to the manufacturer’s instruction. The enriched peptides were used for LC-MS/MS scan on the Q Exactive HF and EASY-nLC 1200 system (Thermo Scientific, Waltham, MA, United States). ProteomeDiscoverer (version 2.4) was used to search raw data against the sample protein database. A global false discovery rate (FDR) was set to 0.01, and protein groups considered for quantification required at least two peptides. The detailed protocol and parameters were set as described in Supplementary Table 2. The amino acid sequences of differentially phosphorylated proteins were used to analyze their Kyoto Encyclopedia of Genes and Genomes (KEGG) pathway and functional enrichment.

### Statistical Analysis

All experiments were performed with three replicates. The data were showed as mean ± SD (standard deviation) and analyzed by using Student’s *t*-test at *P ≤ 0.05 and **P ≤ 0.01.

## Results

### *ZmPP2C26* Is Subject to Alternative First Exon-Type as Generating Two Variants

**FIGURE 1:**
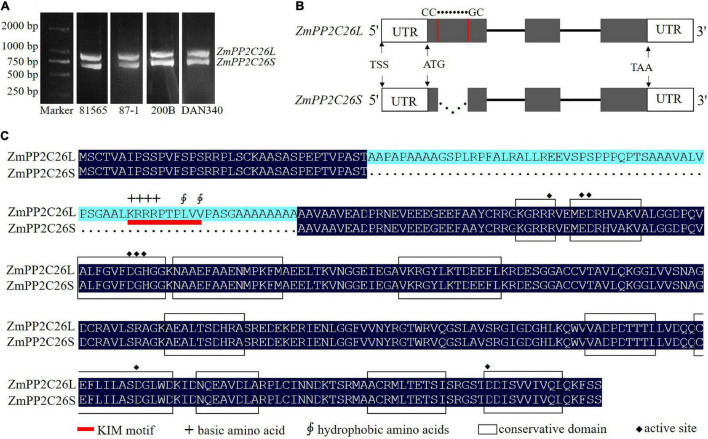
The splicing variants of ZmPP2C26 *via* AFE type. **(A)** The ORF amplification of *ZmPP2C26* gene from maize inbred lines 81565, 87-1, 200B, and DAN340. **(B)** The alternative splicing model of *ZmPP2C26*. The gray boxes indicate exons. Lines correspond to introns; dotted line represents alternative splicing events; UTR, untranslated regions; TSS, the transcriptional start site; The splicing site is 5′-CC● ● ● ● ●●GC-3′. **(C)** Protein sequence alignment of ZmPP2C26L and ZmPP2C26S. Black boxes indicate conservation domain of protein phosphatase; red line indicates KIM motif; +, basic amino acid; ∮, hydrophobic amino acids; ◆, active site.

During gene cloning, it is found that two AS variants were amplified using a pair of *ZmPP2C26* primers and defined as *ZmPP2C26L* and *ZmPP2C26S* (Figure 1A). Sequence alignment showed that there was 213 bp retention in the first exon of *ZmPP2C26L* compared with *ZmPP2C26S*. The splicing site is 5′-CC● ● ● ● ● ●GC-3′ and neither U2 nor U12 type, indicating that it is a new AS-type AFE (Figure 1B and Supplementary Figure 1A). Protein sequence alignment showed that 71 amino acids were encoded by 213 bp nucleotides and possessed a highly conserved MAPK interaction motif (KIM motif, [K/R]-(3-4)-X(1-6)-[L/I]-X-[L/I]) (Figure 1C and Supplementary Figure 1B; Schweighofer et al., 2007), implying that ZmPP2C26L might interact with some ZmMAPK members.

### *ZmPP2C26* Splicing Variants Interacts With ZmMAPK3 and ZmMAPK7

**FIGURE 2:**
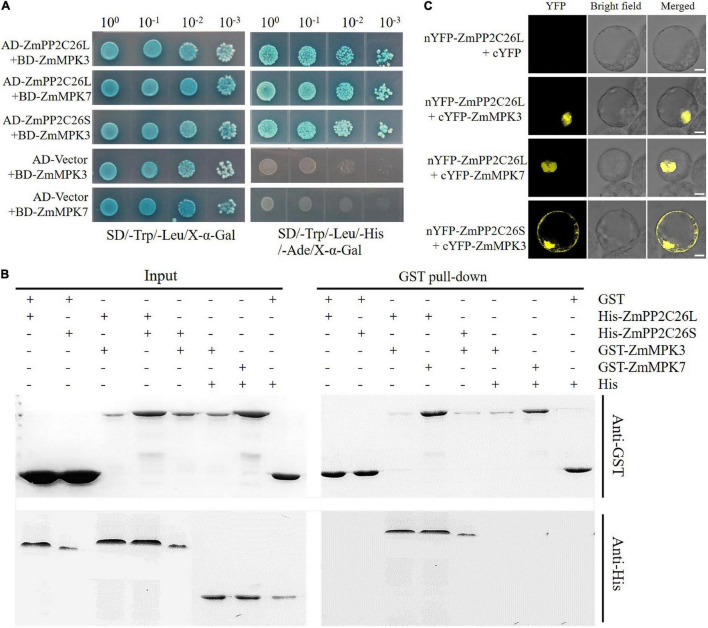
The interaction between ZmPP2C26L/ZmPP2C26S and ZmMAPK3/ZmMAPK7. **(A)** Y2H assay. **(B)** GST pull-down assay. **(C)** BiFC assay. Scale bar = 20 μm.

To address whether ZmPP2C26L/ZmPP2C26S participated in ABA and MAPK signaling, Y2H assay was performed to determine the interaction of them with 13 ZmPYLs and 20 ZmMAPKs. The results showed that ZmPP2C26L/ZmPP2C26S did not interact with 13 ZmPYLs (Supplementary Figure 2A). However, on the quadruple dropout (–Leu/–Trp/–His/–Ade/) SD plates with X-α-Gal, the yeast strains cotransformed by AD-ZmPP2C26L and BD-ZmMAPK3/BD-ZmMAPK7, AD-ZmPP2C26S, and BD-ZmMAPK3, as well as positive control (i.e., AD-T and BD-53) could grow well and be stained blue (Figure 2A and Supplementary Figure 2B). The GST pull-down showed that the His-ZmPP2C26L was pulled down by GST-ZmMAPK3/-ZmMAPK7, and His-ZmPP2C26S was only pulled down by GST-ZmMAPK3 (Figure 2B). The BiFC assay further showed that co-expression of nYFP-ZmPP2C26L and cYFP-ZmMAPK3/cYFP-ZmMAPK7 and co-expression of nYFP-ZmPP2C26S and cYFP-ZmMAPK3 in maize protoplasts could produce strong YFP fluorescence signal (Figure 2C). These results confirm that ZmPP2C26L physically interact with ZmMAPK3 and ZmMAPK7, but ZmPP2C26S only interacts with ZmMAPK3 *in vitro* and *in vivo*.

### *ZmPP2C26* Dephosphorylates ZmMAPK3 and ZmMAPK7

**FIGURE 3:**
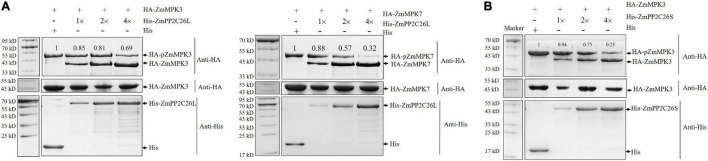
The dephosphorylation assay *in vitro*. **(A)** ZmPP2C26L dephosphorylates ZmMAPK3 and ZmMAPK7. **(B)** ZmPP2C26S dephosphorylates ZmMAPK3. The phospho-ZmMAPK3/-ZmMAPK7 with HA tag was extracted from maize protoplasts, incubated with His-ZmPP2C26L/His-ZmPP2C26S, separated by 12.5% phos-tag SDS-PAGE gel (top panel) or normal SDS-PAGE gel (middle panel), and detected by using an anti-HA or anti-His antibody. Different amounts of His-ZmPP2C26L/His-ZmPP2C26S were used to dephosphorylate ZmMAPK3 and ZmMAPK7. 1×, 2×, and 4× represent 0.25, 0.5, and 1.0 μg purified His-ZmPP2C26L/-ZmPP2C26S, respectively. + and - denote the presence and absence of the protein in each sample, respectively. The relative intensity of the protein bands was measured using ImageJ, and the lane without His-ZmPP2C26L/His-ZmPP2C26S was set to 1.00.

To test whether ZmPP2C26L/ZmPP2C26S dephosphorylates ZmMAPK3/ZmMAPK7, the dephosphorylation assay was performed *in vitro*. As shown in Figure 3, only phospho-ZmMAPK3/-ZmMAPK7 was detected in the absence of ZmPP2C26L or ZmPP2C26S on the phos-tag™ SDS-PAGE gel. However, de-phosphorylated ZmMAPK3/ZmMAPK7 was detected when HA-ZmMAPK3/HA-ZmMAPK7 was incubated with His-ZmPP2C26L and HA-ZmMAPK3 was incubated with His-ZmPP2C26S. Notably, the phospho-ZmMAPK3/phospho-ZmMAPK7 proteins were decreased with the increase in ZmPP2C26L/ZmPP2C26S concentration. Furthermore, only dephospho-ZmPP2C26L/-ZmPP2C26S was detected using anti-His when HA-ZmMAPK3/HA-ZmMAPK7 was incubated with His-ZmPP2C26L and HA-ZmMAPK3 was incubated with His-ZmPP2C26S. These results show that ZmPP2C26L dephosphorylates ZmMAPK3 and ZmMAPK7, and ZmPP2C26S dephosphorylates ZmMAPK3, whereas ZmPP2C26L and ZmPP2C26S cannot be phosphorylated by ZmMAPK3 and ZmMAPK7. Likewise, the expression of *ZmMAPK3* and *ZmMAPK7* gene was dramatically evaluated elevated by drought stress in maize (Supplementary Figure 3).

### Subcellular Localization of ZmPP2C26L/ZmPP2C26S

**FIGURE 4:**
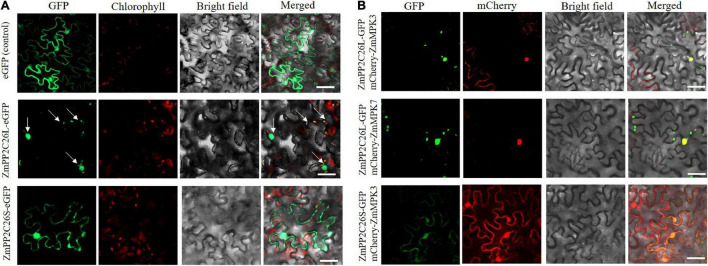
Subcellular localization and co-localization. **(A)** The localization of ZmPP2C26L and ZmPP2C26S. **(B)** Co-localization of ZmPP2C26L and ZmMAPK3/ZmMAPK7 and of ZmPP2C26S and ZmMAPK3. *ZmPP2C26L* and *ZmPP2C26S* were fused with *eGFP*. *ZmMAPK3* and *ZmMAPK7* were separately fused with mCherry. A 4-week-old tobacco (*Nicotiana benthamiana*) seedling was used for infiltrating using *Agro*. Tumefaciens cells harboring above plasmids. Scale bar = 50 μm.

To determine the subcellular localization of ZmPP2C26L/ZmPP2C26S, the ORF of them was fused with *eGFP* under the control of the *35S* promoter and introduced into *N. benthamiana* leaves for transient expression. Confocal laser scanning microscopy showed that the eGFP fluorescence signal was observed in both the cytoplasm and the nucleus from the leaf infiltrated by the empty *eGFP* vector (control) and *ZmPP2C26S-eGFP* vector, but in the chloroplast and nucleus from the leaf infiltrated by the *ZmPP2C26L-eGFP* vector (Figure 4A). Co-localization also showed that ZmPP2C26L co-localized with ZmMAPK3/ZmMAPK7 in the nucleus, and ZmPP2C26S co-localized with ZmMAPK3 in the cytoplasm and nucleus (Figure 4B).

### ZmPP2C26S Exhibits Higher Phosphatase Activity Than ZmPP2C26L

Since ZmPP2C26 is a member of PP2C clade B and belongs to PP family (Wang et al., 2018), the phosphatase activity of ZmPP2C26L and ZmPP2C26S was detected using the chromogenic substrate pNPP *in vitro* phosphatase assays. The PP could catalyze pNPP to produce p-nitrophenol with an absorbance value of OD_405_. After adding the purified His-ZmPP2C26L and His-ZmPP2C26S into the reaction solution, there was a strong OD_405_ values, suggesting that both of them had phosphatase activity. Moreover, the relative phosphatase activity of ZmPP2C26S was significantly higher than ZmPP2C26L (Supplementary Figure 4).

### ZmPP2C26L and ZmPP2C26S Negatively Regulate Drought Tolerance

The results of RT-qPCR showed that the expression of *ZmPP2C26* and *ZmPP2C26L* was significantly downregulated by drought stress in drought-tolerant lines 81565 and 87-1 and upregulated in drought-sensitive lines 200B and DAN340 (Supplementary Figure 5), which was consist with our previous study (Lu et al., 2020). The data imply that *ZmPP2C26* plays a crucial role in regulating drought tolerance.

**FIGURE 5:**
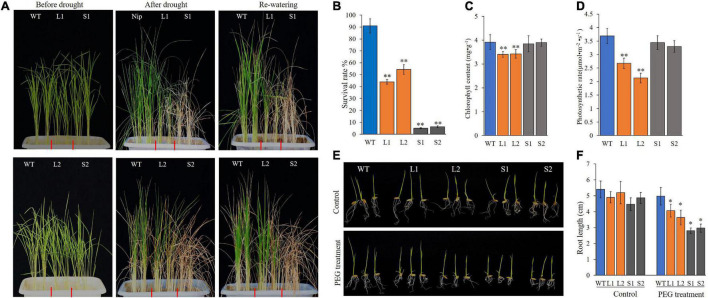
Phenotyping of transgenic rice lines under drought stress. **(A)** Wilting phenotype of every line. Three-week-old seedings were subjected to drought treatment by withholding watering for 2 weeks, then re-watered for 3 days and photographed. **(B)** The survival rate of every line after treatment. **(C,D)** Chlorophyll content and photosynthetic rate of every line before treatment. **(E)** Root phenotype of every line. After germination, seedlings were transferred into plastic net pots and cultured in 20% PEG solution for 3 days. **(F)** Root length. WT, wild type; L1 and L2, *ZmPP2C26L*-overexpressing lines; S1 and S2, *ZmPP2C26S*-overexpressing lines. *P < 0.05; **P < 0.01.

Hence, *ZmPP2C26L* and *ZmPP2C26S* was complemented and overexpressed in *Arabidopsis*-mutant *ap2c1* (AT2G30020, an ortholog of *ZmPP2C26*) and WT. The complementation and overexpression of *ZmPP2C26L*/*ZmPP2C26S* increased the drought sensitivity of transgenic lines compared with *ap2c1* and WT (Supplementary Figure 6). Subsequently, they were further overexpressed in rice (*Nipponbare*). Two *ZmPP2C26L*-overexpressing lines (i.e., L1 and L2) and two *ZmPP2C26S*-overexpressing lines (i.e., S1 and S2) were subjected to drought stress by withholding watering. As shown in Figure 5, after 2 weeks of drought stress, transgenic lines showed enhancement of drought sensitivity, but WT seedlings were slightly blasted. The survival rates of L1, L2, S1, and S2 lines were 44.5, 54.3, 5.2, and 6.0%, respectively, which were significantly lower than WT (90.8%). Furthermore, the root length of transgenic lines was significantly shorter than WT under drought stress condition. Under optimal condition, the chlorophyll content and photosynthetic rates of L1 and L2 lines were significantly lower than WT. S1 and S2 lines showed no significant difference compared with WT.

**FIGURE 6:**
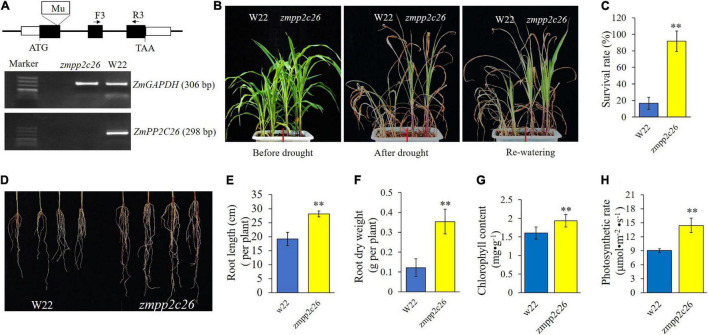
Phenotyping of maize *zmpp2c26* under drought stress. **(A)** Schematic diagram of Mu transposon in *ZmPP2C26* gene and transcriptional detection *ZmPP2C26* by RT-PCR. Black boxes represent exons; F and R, primer position in *ZmPP2C26*, and the sequences of them were listed in Supplementary Table 1. A 298 bp fragment of *ZmPP2C26* was amplified from *zmpp2c26* cDNA using F and R primers. A 306 bp fragment of *ZmGAPDH* was amplified and used as reference. W22, the wild type isolated from heterozygous *zmpp2c26* mutant, was used as control. **(B)** Wilting phenotype of every line. The three-leaf stage seedlings were subjected to drought stress *via* withholding watering for 3 weeks, then re-watered for 5 days and photographed. **(C)** The survival rate of every line after treatment. **(D)** Root phenotype of every line. **(E,F)** Root length and root dry weight of every line. **(G,H)** Chlorophyll content and photosynthetic rate of every line before treatment. **P < 0.01.

Meanwhile, a maize mutant of *zmpp2c26* was used for drought tolerance test. As shown in Figure 6, the Mu transposon insertion of *zmpp2c26* resulted in knockout of *ZmPP2C26* identified by RT-PCR. The *zmpp2c26* exhibited drought-tolerant phenotype compared with WT. After drought stress, the survival rate of *zmpp2c26* was 87.5% and that of the WT was only 12.5%. The root length and root dry weight of *zmpp2c26* were also significantly higher than WT. The chlorophyll content and photosynthetic rate of *zmpp2c26* mutant were significantly higher than WT. The above results suggest that *ZmPP2C26* negatively modulates drought tolerance and the *ZmPP2C26S* variant was more sensitive to drought than *ZmPP2C26L*.

### ZmPP2C26 Alters Protein Phosphorylation Level

**FIGURE 7:**
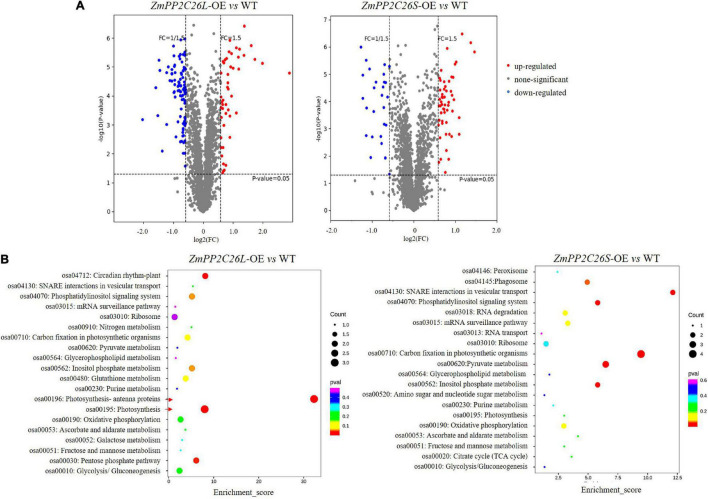
Phosphoproteomics of transgenic rice identifies a set of *ZmPP2C26*-affected phosphoproteins. **(A)** Phosphopeptide abundance for *ZmPP2C26L*- and *ZmPP2C26S*-overexpressing lines compared with WT. **(B)** The GO and KEGG analysis of *ZmPP2C26L*- and *ZmPP2C26S*-affected phosphorylation proteins.

To uncover the global effects of ZmPP2C26 on protein phosphorylation, we performed TMT-based quantitative phosphoproteomic analysis in the *ZmPP2C26L*-/*ZmPP2C26S*-overexpressing lines and WT. A total of 3,516 phosphosites and 2,877 phosphopeptides derived from 1,532 phosphoproteins with >1.5-fold change (*P* < 0.05) were found to form transgenic lines compared with WT (Supplementary Table 3). After evaluation of raw data, clean data with high reliability were screened and used for KEGG analysis. Compared with WT, 48 and 93 phosphopetides were significantly upregulated and downregulated in the *ZmPP2C26L*-overexpression lines, respectively. In contrast, 55 and 28 phosphopetides were significantly upregulated and downregulated in the *ZmPP2C26S*-overexpression lines, respectively (Supplementary Table 4). KEGG enrichment pathway analysis showed that the *ZmPP2C26L*-affected phosphorylation proteins predominately enriched in photosynthesis and *ZmPP2C26S*-affected phosphorylation proteins were associated with carbon fixation in photosynthesis and pyruvate metabolism (Figure 7). Among them, 8 upregulated and 12 downregulated phosphoproteins were shared by *ZmPP2C26L-OE* and *ZmPP2C26S-OE* lines compared with WT, and the aquaporin PIP2-7 showed the highly increased phosphorylation level (Supplementary Table 5).

**FIGURE 8:**
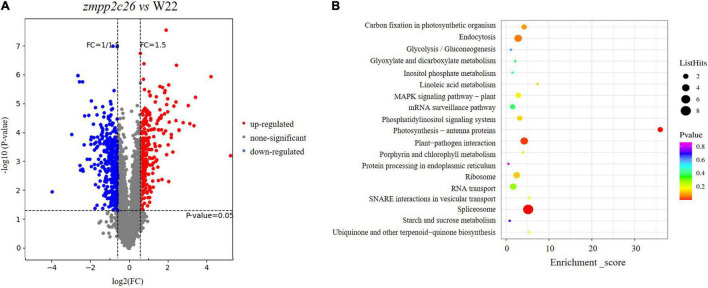
Phosphoproteomics of *zmpp2c26* mutant identifies *ZmPP2C26*-affected phosphoproteins. **(A)** Phosphopeptide abundance for *zmpp2c26* compared with WT. **(B)** The KEGG analysis of *ZmPP2C26*-affected upregulated phosphorylation proteins.

Similarly, we analyzed the phosphoproteomic difference of *zmpp2c26* compared with WT. The results showed that a total of 671 unique phosphorylation proteins with >1.5-fold change (*P* < 0.05) were identified in *zmpp2c26* (Supplementary Table 6). Also, 324 and 347 phosphoproteins were upregulated and downregulated in *zmpp2c26*, respectively. As ZmPP2C26 is a PP, the phosphorylation level of its targets should be increased in the *zmpp2c26* plants. The KEGG pathway analysis indicated that the upregulated phosphoproteins were enriched in several pathways but mainly involved in photosynthesis (Figure 8). Notably, the phosphorylation level of ZmMAPK3 (accession number: B4FN55) was significantly upregulated in *zmpp2c26* mutant (Supplementary Table 6), indicating that ZmPP2C26 involved in photosynthesis and MAPK-mediated signaling pathway.

## Discussion

Alternative splicing is a critical gene post-transcriptional regulation mechanism for plants in response to surrounding stress, growth, and development (Reddy et al., 2013; Qin et al., 2017). In plants, more than 60% intron-containing genes undergo AS to produce a vast repertoire mRNA isoform (Marquez et al., 2012; Reddy et al., 2013; Iniguez et al., 2017; Jiang et al., 2017). U2 and U12 are two typical intron retention types of AS (Sharp and Burge, 1997; Reddy et al., 2013). In this study, *ZmPP2C26*, a maize PP2C clade B member, is found to undergo AS, producing *ZmPP2C26L* and *ZmPP2C26S* in different maize germplasm lines (Figure 1). Its splicing site is 5′-CC● ● ● ● ●●GC-3′ (Figure 1B and Supplementary Figure 1A), which is AFE-type AS and recently also found in *Arabidopsis* (Wang et al., 2015; Qin et al., 2017; Zhu et al., 2017).

The clade B PP2Cs can recognize MAPK phosphatases and interact with MAPK3, MAPK4, or MAPK6 to regulate stomatal aperture, seed germination, and stress response (Brock et al., 2010; Umbrasaite et al., 2010; Sidonskaya et al., 2016; Shubchynskyy et al., 2017). It has been reported that AP2C1 dephosphorylates CBL-interacting protein kinase (CIPK) to regulate K^+^ deficiency response (Singh et al., 2018). In this study, it is confirmed that 213 bp is retained in first exon of *ZmPP2C26L* and spliced in *ZmPP2C26S*, and encodes 71 amino acids including a conserved KIM motif (Figure 1C and Supplementary Figure 1B). However, the ZmPP2C26L physically interacts with ZmMAPK3 and ZmMAPK7 in the nucleus, but ZmPP2C26S only interacts with ZmMAPK3 in both the cytoplasm and the nucleus (Figures 2, 4B and Supplementary Figure 2B), which means that the molecular mechanism of these two variants may be different. Considering ZmMAPK3 and ZmMAPK7 can be phosphorylated by ZmMKK10 (Chang et al., 2017), they are transiently expressed in maize protoplast, immunoprecipitated, and used for dephosphorylation catalyzed by ZmPP2C26. It is expectedly found that ZmPP2C26L dephosphorylates ZmMAPK3 and ZmMAPK7, and ZmPP2C26S dephosphorylates ZmMAPK3, while ZmMAPK3 and ZmMAPK7 cannot phosphorylate ZmPP2C26L and ZmPP2C26S (Figure 3). The ZmPP2C26L/ZmPP2C26S did not interact with 13 PYLs (Supplementary Figure 2), suggesting that ZmPP2C26L and ZmPP2C26S may act on MAPK signaling to regulate downstream signaling in ABA-independent way. Interestingly, ZmPP2C26S lacks KIM motif but interacts with ZmMAPK3, indicating that KIM motif is unnecessary for interaction between ZmMAPKs and other protein. Meanwhile, *ZmMAPK3* and *ZmMPK7* were found to be upregulated by drought stress (Supplementary Figure 3) and involved in multiple stresses response in previous reports (Wang J. et al., 2010, Wang Y. L. et al., 2010; Wang et al., 2011; Liu et al., 2013). Their homologs, i.e., AtMPK3 and AtMPK6, regulated salt and cold tolerance and hypoxia signaling in *Arabidopsis* (Li et al., 2017; Yan et al., 2021; Zhou et al., 2021). These studies imply that ZmPP2C26 functions in plants stress response acting on ZmMAPK3/ZmMAPK7.

Since GC content flaking splicing sites is pretty high for primer PP2C26S-F (Supplementary Figure 1), fragment of *ZmPP2C26S* was not amplified using primers PP2C26S-F/PP2C26S-R for RT-qPCR, so we detected the relative expression level of *ZmPP2C26* containing the transcript of *ZmPP2C26L* and *ZmPP2C26S* instead of *ZmPP2C26S*. Under drought stress, the expression of *ZmPP2C26* and *ZmPP2C26L* was significantly downregulated by drought stress in drought-tolerant maize and upregulated in drought-sensitive maize (Supplementary Figure 5). The possible cue is due to the inhibition of its promoter by drought (Lu et al., 2020). The results indicate that *ZmPP2C26* may play a negative role in regulating drought tolerance. Hence, they are functional validated *via* ectopic expressing in *Arabidopsis*, rice, as well as phenotyping maize *zmpp2c26* mutant (Figures 5, 6 and Supplementary Figure 6). But the complementation of *ZmPP2C26L* in *Arabidopsis ap2c1* mutant partially restores drought-sensitive phenotype and overexpression of *ZmPP2C26L* in *Arabidopsis* WT and rice exhibit much lower hypersensitive to drought tolerance compared with *ZmPP2C26S*-overexpression lines (Figure 5 and Supplementary Figure 6). These results may be explained by ZmPP2C26S possesses higher activity compared with ZmPP2C26L (Supplementary Figure 4). The mutant of *ZmPP2C26* gene increases chlorophyll and photosynthesis rate (Figures 6G,H), which could be explained by affecting the phosphorylation of proteins involved in photosynthesis (Figure 8). However, the downstream of ZmMAPK3- and ZmMAPK7-meditated by ZmPP2C26 needs to be explored in further study. In transgenic rice, ZmPP2C26L affects proteins phosphorylation directly enriched in photosynthesis, but ZmPP2C26S regulates proteins associated with carbon fixation in photosynthesis and pyruvate metabolism (Figure 7), and the phosphorylation of aquaporin PIP2-7 contributes for drought tolerance influenced by ZmPP2C26L and ZmPP2C26S (Jang et al., 2014; Fan et al., 2015).

## Conclusion

**FIGURE 9:**
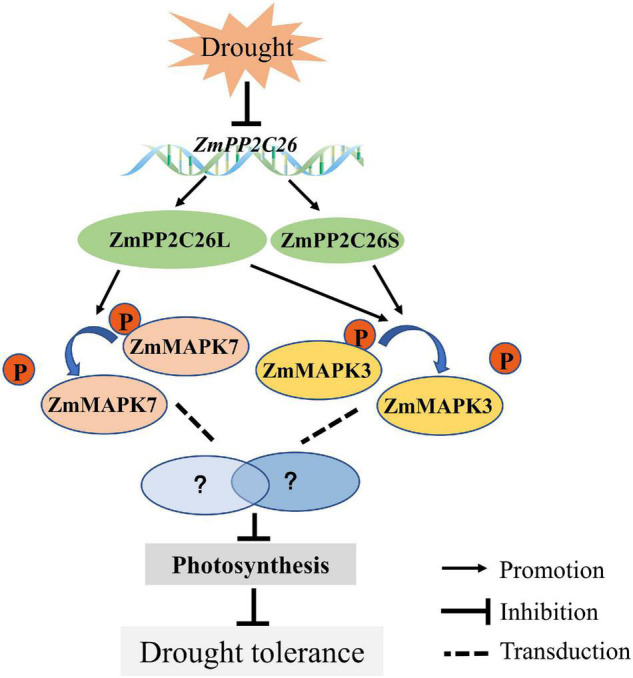
The proposed model depicting ZmPP2C26 alternative splice variants negatively regulate drought tolerance.

We characterized a clade B type 2C PP, i.e., ZmPP2C26, that undergoes untypical AS to generate two isoforms. ZmPP2C26 directly dephosphorylates ZmMAPK3 and ZmMAPK7 to negatively regulate drought stress and photosynthesis activity (Figure 9). This study provides insights into understanding the mechanism of PP2C in response to abiotic stress.

## Data Availability Statement

The original contributions presented in the study are included in the article/Supplementary Material, further inquiries can be directed to the corresponding author/s.

## Author Contributions

FL performed most of the experiments and drafted the manuscript. FL and HY designed the experiments. YP, YC, JQ, and FS carried out the experiments and analyzed the data. LZ and QY assisted in experimental operations. XZ, YL, and FF reviewed the manuscript. WL and HY edited the manuscript. All authors read and approved the final manuscript.

## Conflict of Interest

The authors declare that the research was conducted in the absence of any commercial or financial relationships that could be construed as a potential conflict of interest.

## Publisher’s Note

All claims expressed in this article are solely those of the authors and do not necessarily represent those of their affiliated organizations, or those of the publisher, the editors and the reviewers. Any product that may be evaluated in this article, or claim that may be made by its manufacturer, is not guaranteed or endorsed by the publisher.

## Footnotes

1
https://imagej.en.softonic.com/

2
http://chinamu.jaas.ac.cn/Default.html
