# Supplementary material for: ZmPP2C26 Alternative Splicing Variants Negatively Regulate Drought Tolerance in Maize
Source: Front Plant Sci. 2022 Apr 8;13:851531. doi: 10.3389/fpls.2022.851531 (PMC9024303; doi:10.3389/fpls.2022.851531)

**SUPPLEMENTARY FIGURE 1.** Sequence alignments. (A) The alignment of coding sequence of *ZmPP2C26L* and *ZmPP2C26S*. PP2C26S-F/PP2C26S-R, PP2C26L-F/PP2C26L-R and PP2C26-F/PP2C26-R indicates primers used for amplification of *ZmPP2C26S*, *ZmPP2C26L* and *ZmPP2C26* fragment during RT-qPCR, respectively. (B) The alignment of 71 amino acids between N-terminal of clade B PP2Cs of *Arabidopsis*. Red box represents KIM motif; +, basic amino acid;∮, hydrophobic amino acids.


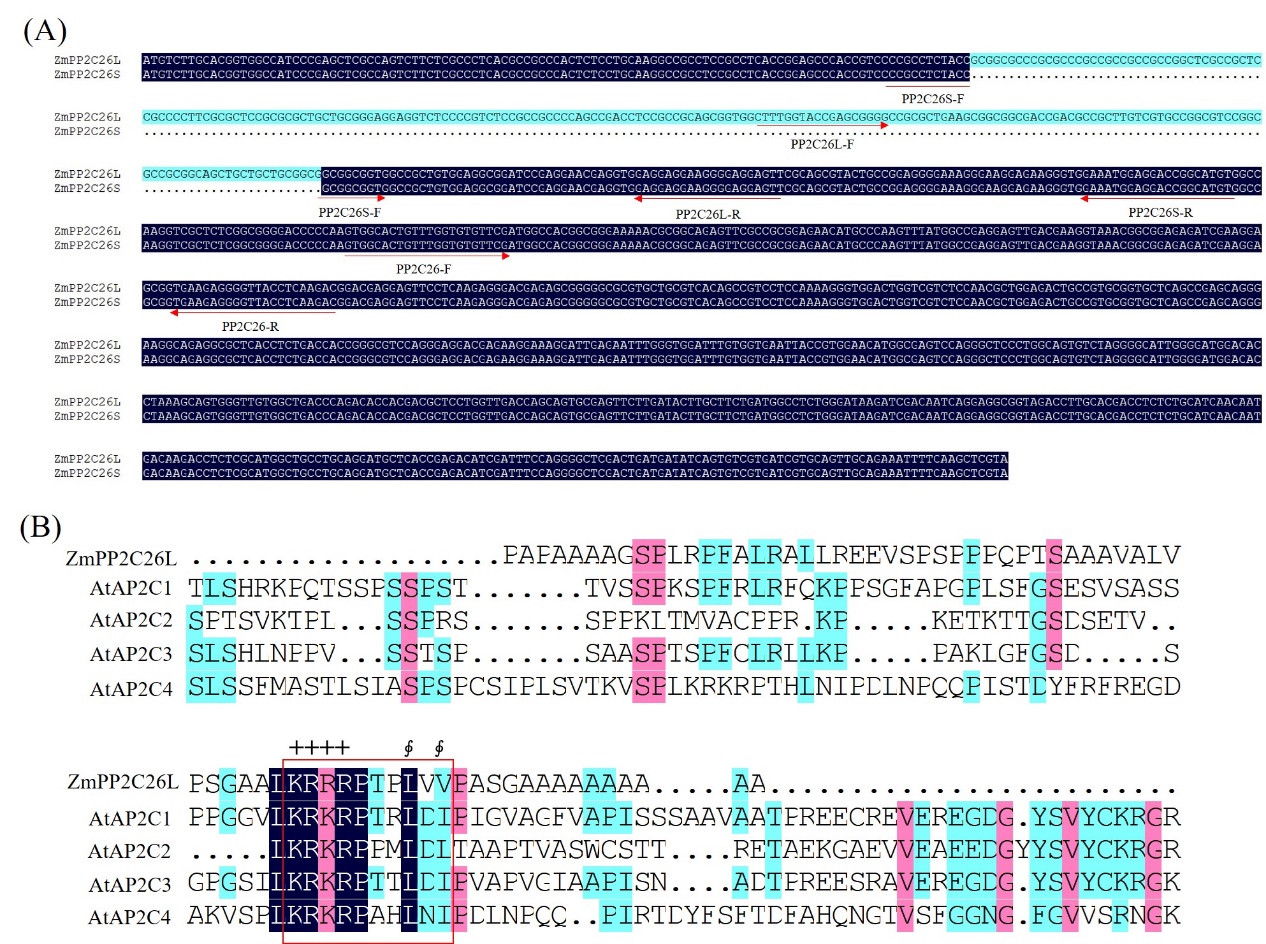


**SUPPLEMENTARY FIGURE 2.** Y2H between ZmPP2C26L/ZmPP2C26S and 13 ZmPYLs (A) and 20 ZmMAPKs (B). DDO/X means double dropout (-Leu/-Trp) SD plates with X-α-Gal, QDO/X represents quadruple dropout (-Ade/-His/-Leu/-Trp) SD plates with X-α-Gal.**
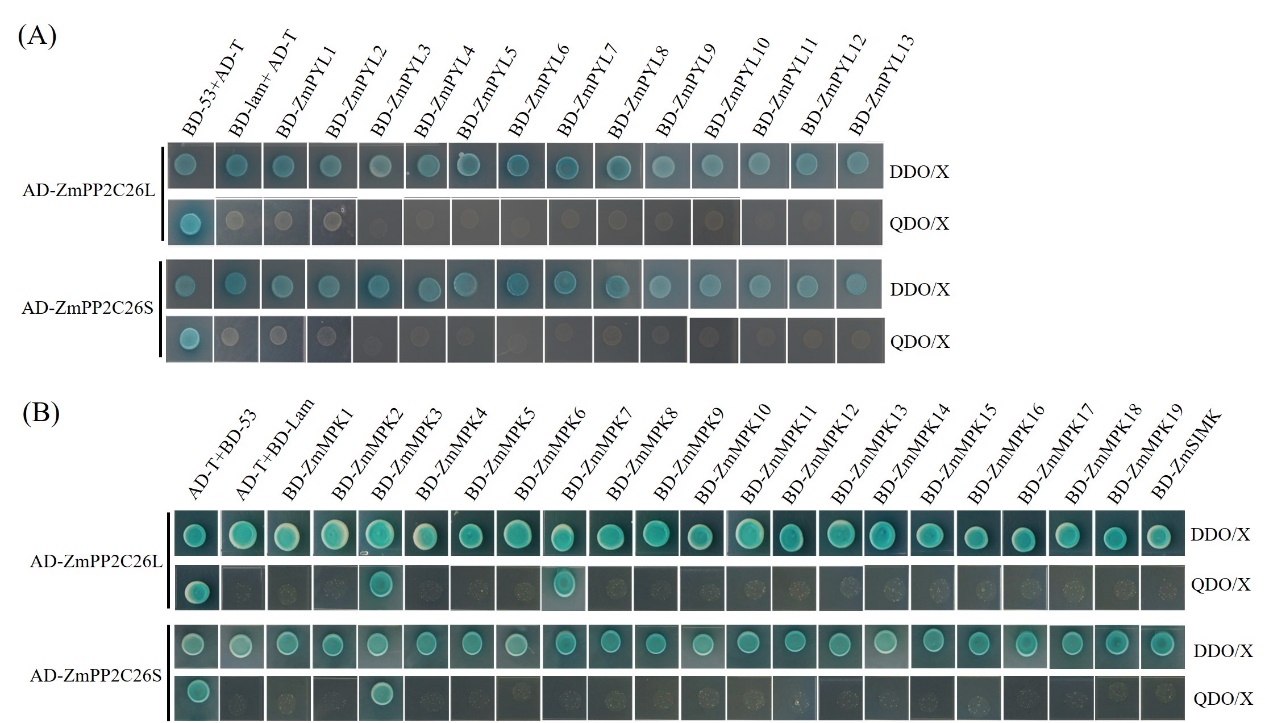
**

**SUPPLEMENTARY FIGURE 3.** The relative expression level of *ZmMPK3* and *ZmMPK7* gene under mimic drought stress. The maize seedlings of inbred lines 87-1 and B73 were treated by 16%-PEG for 0 (control), 3, 6,12 and 24 h. Data represent the means ± SD of three biological replicates. *, P < 0.05; **, P< 0.01; ***, P< 0.001.


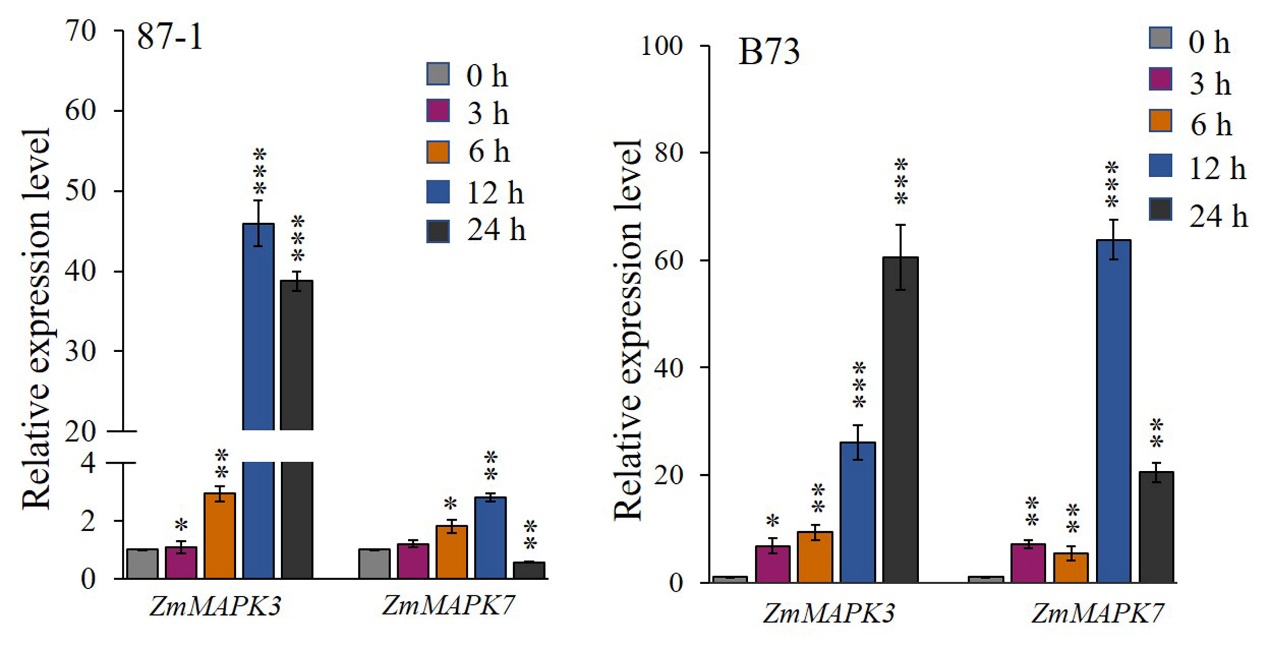


**SUPPLEMENTARY FIGURE 4.** Phosphatase activity of ZmPP2C26L and ZmPP2C26S. (A) Curve of t-OD_405_. Absorbance value at 405 nm of p‑nitrophenol produced from pNPP under the catalyzation by ZmPP2C26L/ZmPP2C26S was monitored every 2 min. (B) The relative phosphatase activity of ZmPP2C26L and ZmPP2C26S. The enzyme activities were calculated based on the curve of t-OD_405_.


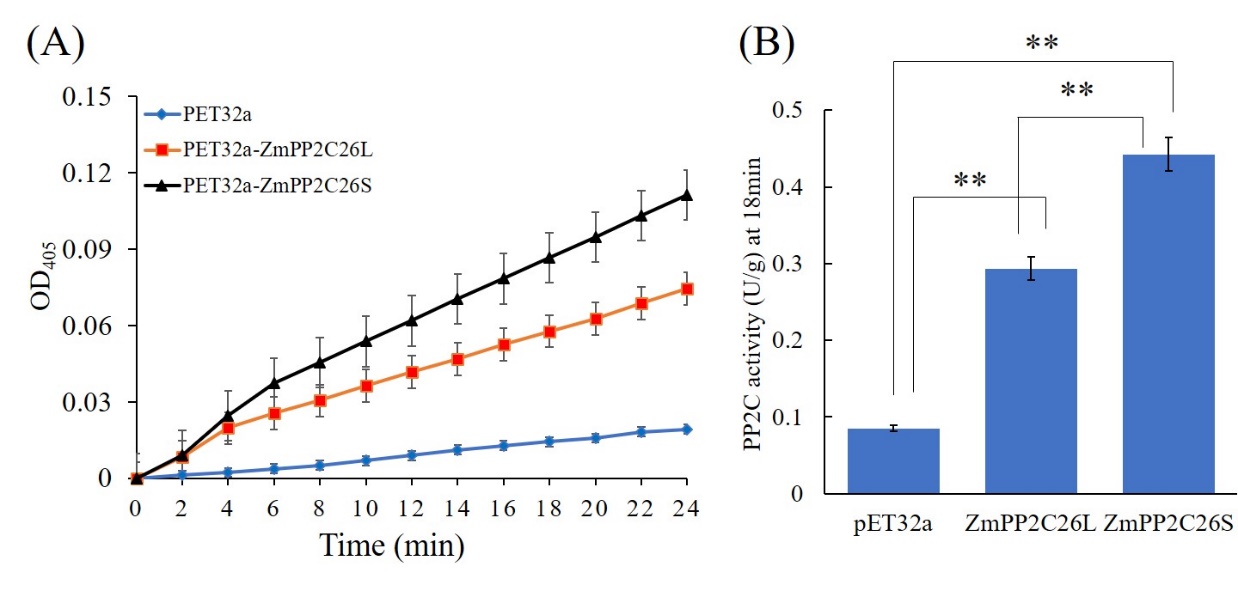


**SUPPLEMENTARY FIGURE 5.** The relative expression level of *ZmPP2C26* under mimic drought stress. The maize seedlings of drought-tolerant inbred lines 81565/87-1 and drought-sensitive lines 200B/DAN340 were treated by 16%-PEG for 0 (control), 3, 6,12 and 24 h. Data represent the means ± SD of three biological replicates. *, P < 0.05; **, P< 0.01.

**
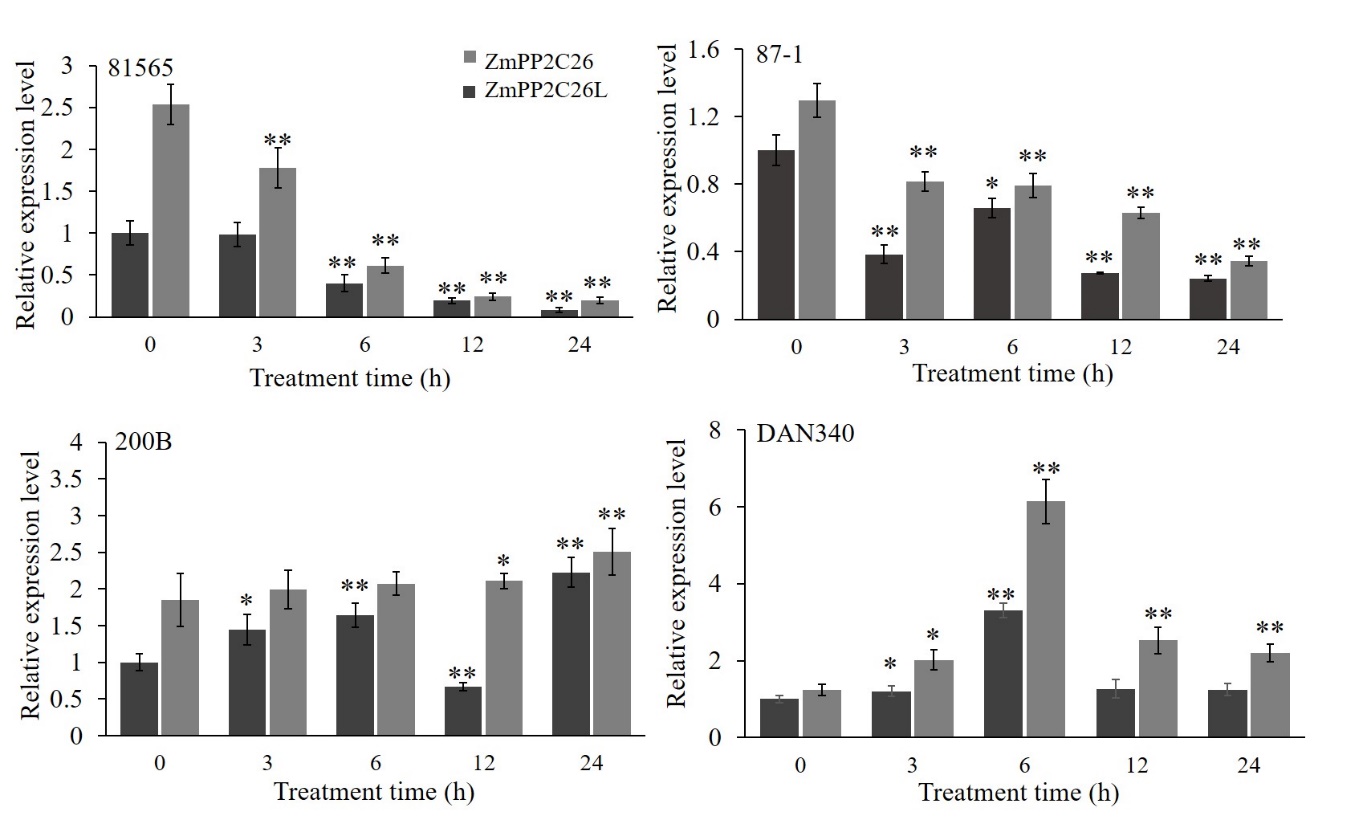
**

**SUPPLEMENTARY FIGURE 6.** Phenotype of transgenic *Arabidopsis* lines under drought stress. (A) Complementation assay. The *ZmPP2C26L* and *ZmPP2C26S* were transformed into *Arabidopsis ap2c1* mutant, respectively. *ap2c1*, untransformed mutant; WT, untransformed wild type; RE#L1 and RE#L2, *ZmPP2C26L* complementary lines in *ap2c1* background; RE#S1 and RE#S2, *ZmPP2C26S* complementary lines in *ap2c1* background. (C) Overexpression assay. The *ZmPP2C26L* and *ZmPP2C26S* were transformed into *Arabidopsis* wild type (WT, Col-0 background), respectively. OE#L1 and OE#L2, *ZmPP2C26L* overexpressing lines in WT background; OE#S1 and OE#S2, *ZmPP2C26S* overexpressing lines in WT background. Four-week-old seedlings were kept under water deprivation for two weeks, then re-watered with a recovery time two days.


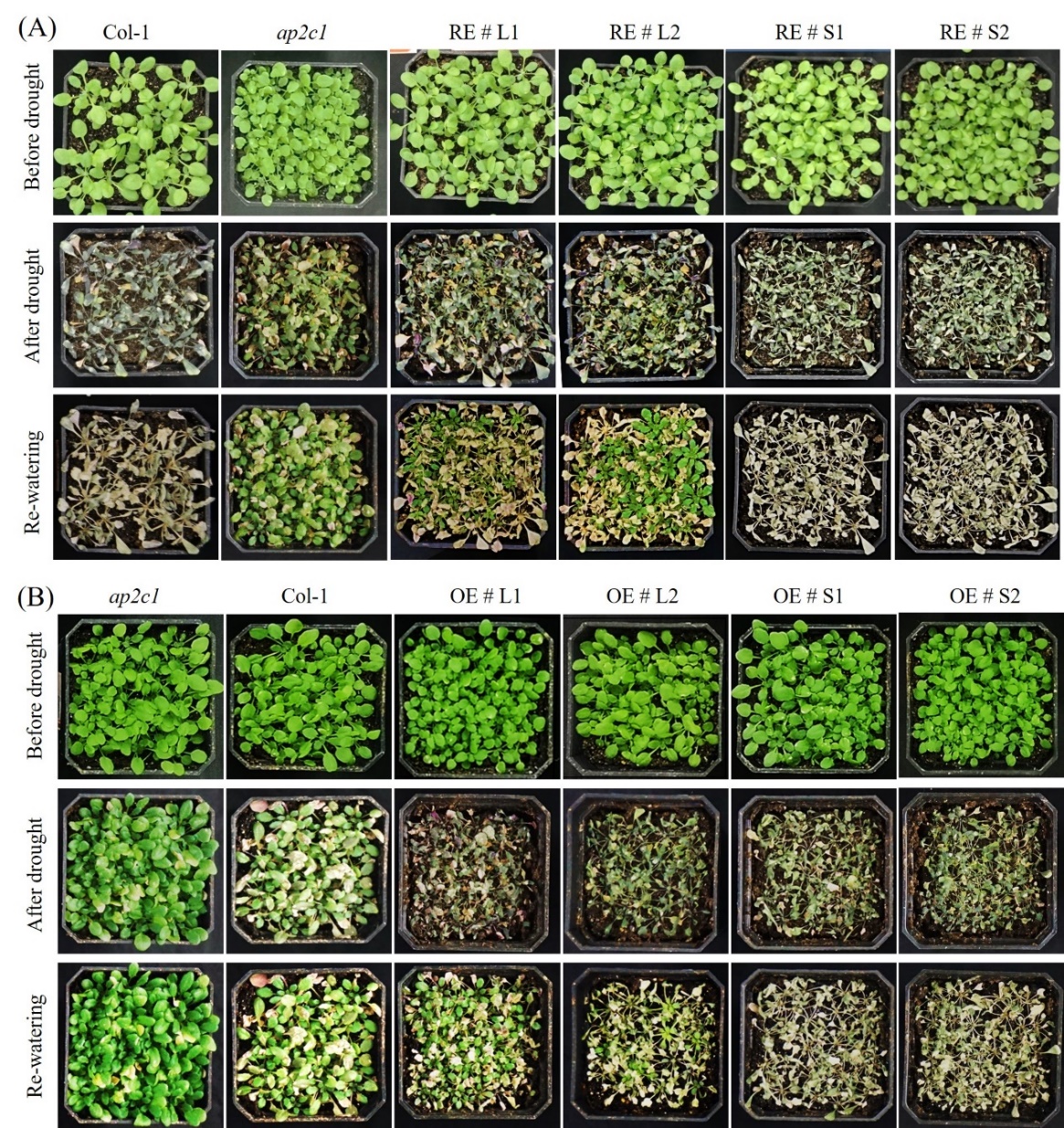

Supplement: Supplementary file 2 [file Data_Sheet_1.DOCX]
