# Supplementary material for: ZmPP2C26 Alternative Splicing Variants Negatively Regulate Drought Tolerance in Maize
Source: Front Plant Sci. 2022 Apr 8;13:851531. doi: 10.3389/fpls.2022.851531 (PMC9024303; doi:10.3389/fpls.2022.851531)
Supplement: Supplementary file 3 [file Data_Sheet_3.DOCX]

**The protocol of TMT-labeling Quantitative Phosphoproteomics**

The leaf samples were frozen with liquid nitrogen and fully ground. The powdered tissue was suspended in 1 ml extract buffer (1.5 M sucrose, 0.1M NaCl, 0.05 M EDTANa_2_, 0.015 M DTT, 0.125 M Tris-HCl (pH 6.8), 0.125 M Tris-HCl (pH8.8)) centrifuged at 7100 g for 5 min at 4 ℃. Added 5 volumes of pre-chilled 0.1M Ammonium Acetate-Methanol solution, and precipitated overnight at -20°C. Centrifuged at 12000 g for 10 min at 4 °C to collected precipitate. Washed precipitate with acetone and methanol respectively. Collected precipitate and lysed in lysis buffer (6M guanidine hydrochloride in 100mM Tris-HCl, pH 8.5) with 10mM NaF, EDTA-free protease for 3 h at room temperature, centrifuged at 12000g for 10 min to take the supernatant, and then added phosphatase inhibitor cocktails (Roche, Swiss). The extracted protein amount was quantified using the BCA assay (Thermo Fisher Scientific, USA). Add DTT (5 mM) and iodotyrosine (10 mM) to 200 μg protein samples, and place them in the dark at room temperature for 15 minutes. Added 6 volumes of acetone to precipitate the protein, and leave it at -20°C for more than four hours or overnight. Centrifuged at 8000 × g for 10 minutes at 4°C to collect the precipitate, and volatilize the acetone for 2-3 minutes. Added 100 μL of TEAB (200 mM) to reconstitute the pellet, add 1/50 of the sample weight of 1 mg/ml Trypsin-TPCK, and digested at 37°C overnight.

Labelled peptides using TMT Labeling Kit (Thermo Fisher Scientific, USA) according to the manufacturer’s instructions. Phosphopeptide enrichment was performed according to the IMAC Phosphopeptide Enrichment Kit (Thermo Fisher Scientific, USA) instructions.

All samples were loaded onto the pre-column Acclaim PepMap100 100μm ⅹ 2cm (RP-C18, Thermo Fisher, USA) at a flow rate of 300 nL/min, and then separated by the analytical column Acclaim PepMap RSLC, 75 μm×15cm (RP-C18, Thermo Fisher, USA). Full MS scans were acquired in the mass range of 350 – 1500 m/z with a mass resolution of 60000 and the AGC target value was set at 3e6. The 20 most intense peaks in MS were fragmented with higher-energy collisional dissociation (HCD) with collision energy of 30. MS/MS spectra were obtained with a resolution of 15000 with an AGC target of 2e5 and a max injection time of 40 ms. The Q Exactive HF dynamic exclusion was set for 30.0 s and run under positive mode.

ProteomeDiscoverer (v.2.4) was used to search all of the Q Exactive raw data thoroughly against the sample protein database. Database search was performed with Trypsin digestion specificity. Alkylation on cysteine was considered as fixed modifications in the database searching. For protein quantification method, TMT labeling was selected. A global false discovery rate (FDR) was set to 0.01 and protein groups considered for quantification required at least 2 peptides.
